# Protective Role for Antioxidants in Acute Kidney Disease

**DOI:** 10.3390/nu9070718

**Published:** 2017-07-07

**Authors:** Joanne M. Dennis, Paul K. Witting

**Affiliations:** Redox Biology Group, Discipline of Pathology, Charles Perkins Centre, Sydney Medical School, The University of Sydney, Sydney, NSW 2006, Australia; jo-dennis@optusnet.com.au

**Keywords:** antioxidant, renal injury, oxidant, hypoxia, ischemia, vitamin C, endothelium

## Abstract

Acute kidney injury causes significant morbidity and mortality in the community and clinic. Various pathologies, including renal and cardiovascular disease, traumatic injury/rhabdomyolysis, sepsis, and nephrotoxicity, that cause acute kidney injury (AKI), induce general or regional decreases in renal blood flow. The ensuing renal hypoxia and ischemia promotes the formation of reactive oxygen species (ROS) such as superoxide radical anions, peroxides, and hydroxyl radicals, that can oxidatively damage biomolecules and membranes, and affect organelle function and induce renal tubule cell injury, inflammation, and vascular dysfunction. Acute kidney injury is associated with increased oxidative damage, and various endogenous and synthetic antioxidants that mitigate source and derived oxidants are beneficial in cell-based and animal studies. However, the benefit of synthetic antioxidant supplementation in human acute kidney injury and renal disease remains to be realized. The endogenous low-molecular weight, non-proteinaceous antioxidant, ascorbate (vitamin C), is a promising therapeutic in human renal injury in critical illness and nephrotoxicity. Ascorbate may exert significant protection by reducing reactive oxygen species and renal oxidative damage via its antioxidant activity, and/or by its non-antioxidant functions in maintaining hydroxylase and monooxygenase enzymes, and endothelium and vascular function. Ascorbate supplementation may be particularly important in renal injury patients with low vitamin C status.

## 1. Introduction

Acute kidney injury (AKI, also known as acute renal failure) is an increasing healthcare challenge [1]. It is defined as a sudden reduction in renal function or glomerular filtration rate (GFR), leading to azotemia and/or insufficient urine production caused by reduced renal blood flow, and kidney damage, inflammation, or obstruction. Clinical presentation of AKI can be wide-ranging, and risk factors include peripheral artery disease, hypertension and diabetes. It is an important cause of morbidity and mortality, and a common complication of traumatic physical injury, sepsis, severe burns, and complex surgery. The socioeconomic importance of AKI is rising, as it is recognized to increase the risk of chronic or end-stage kidney disease, or adverse complications in non-renal tissues such as heart and lung [2].

The majority of AKI causes are associated with ischemia and acute hypoxia from general or regional decreases in renal blood flow. Ischemia severely limits cellular oxygen and nutrient uptake, resulting in acute tubular necrosis and inflammation that can exacerbate renal injury and cause functional changes in the kidney [3]. Ischemia and reperfusion are well-known activators of tissue damage via reactive oxygen species (ROS) [4]. Although ROS perform physiological functions, supra- or unregulated ROS accumulation can cause biomolecule oxidative damage, and perturbations in membrane, macromolecule, and organelle functionality. Detoxification or decomposition processes facilitated by endogenous antioxidants normally counterbalance oxidant production. However, pathophysiological conditions can enhance ROS production and overwhelm the availability and/or decrease endogenous antioxidant activity and promote vascular dysfunction, inflammation, and renal tubule cell cytotoxicity typically observed in the pathogenesis of acute kidney injury (AKI) [5].

Although the exact mechanism whereby ROS are generated in AKI is not defined, decreased ROS generation and oxidative damage are potential therapeutic end points. Antioxidants have the potential to intervene early in the pathogenesis of kidney injury by directly eliminating ROS or the oxidant source. Studies in renal cells, kidney tubules, and animal models of AKI, have identified reno-protective agents with antioxidant activities that mitigate renal oxidative damage (see reviews [5,6,7,8]). This review will focus on oxidative stress in AKI and the therapeutic potential of antioxidants, including the nutrient vitamin C, in experimental and human acute renal injury.

## 2. Oxidative Damage in Acute Renal Injury and Disease

There is considerable evidence that oxidative damage to tubular cells and renal tissue is linked to AKI. Animal studies demonstrate increased oxidative damage and decreased tissue antioxidant status after renal ischemia and/or nephrotoxicity [9,10]. Studies in critically ill or sepsis patients with kidney injury and varying degrees of renal insufficiency, show increased circulating biomarkers of protein and lipid oxidation that correlate with markers of pro-inflammatory, pro-oxidative mediators, and cytokines [11]. Moreover, uremia is associated with increased circulating carbonyl and indole compounds, with the potential to increase systemic oxidative stress [12]. Further, oxidative stress and reactive oxygen species (ROS) are thought to be driving factors in other chronic diseases such as cardiovascular disease and diabetes, that predispose to AKI or are present as co-morbidities in the same subjects [5].

**Figure 1:**
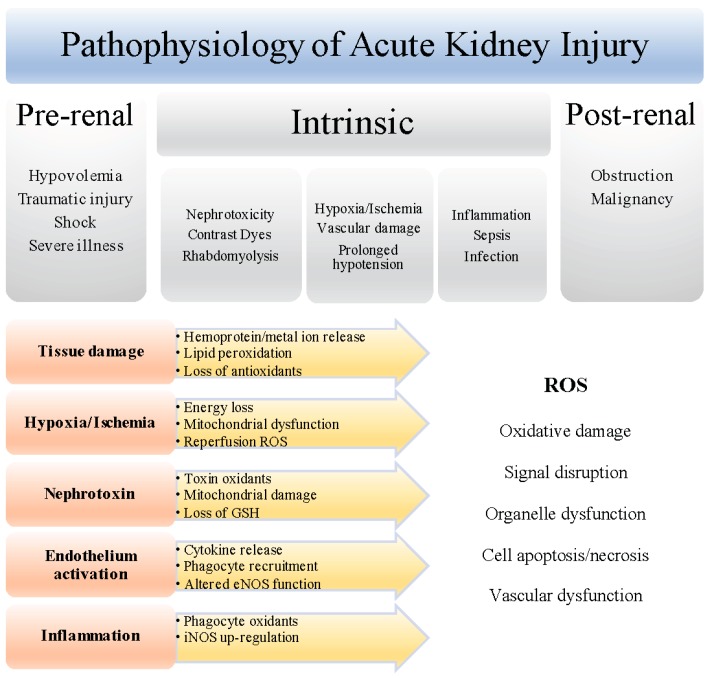
Increased reactive oxygen species (ROS) levels in acute kidney injury (AKI) induce renal oxidative damage and injury. Hypoxia and ischemia perturb microcirculation, cellular enzymes, and mitochondrial function, supporting production of intracellular ROS such as O_2_^•−^ and H_2_O_2_, resulting in mitochondrial damage, depletion of ATP, and activation of cell death pathways. Reperfusion after ischemia also increases ROS. Ischemic injury activates endothelial cells up-regulating pro-inflammatory cytokines and recruiting phagocytes that contribute ROS via NOX and MPO. Inflammation induces ROS and iNOS, promoting peroxynitrite formation. Trauma and toxins generate oxidative stress by depleting endogenous antioxidants and increasing redox-active metal ions. Vascular dysfunction promoted by ischemia, inflammation, or toxicity, affects eNOS function, inducing ROS generation. ROS perturb kinase/phosphatase activities and transcription factor signaling pathways important in cell homeostasis. Oxidative modification of membranes and proteins disrupts cell ion and nutrient transport, energy metabolism, and organelle function, ultimately affecting kidney viability.

Chronic reduction in renal blood flow from pre-existing medical conditions such as liver and renal disease, atherosclerosis, hypertension, diabetes, or from severe illness (traumatic injury, heart failure, sepsis, rhabdomyolysis), or localized acute blood flow insufficiency due to renal ischemia or nephrotoxicity, are responsible for the majority of AKI cases, and primarily manifest as acute obstruction that prevents urine flow [13,14]. The importance of ROS in the pathogenesis of AKI has been intensely examined because hypoxia and ischemia, that link to renal injury, can induce ROS [4] (Figure 1). The kidney is highly sensitive to hypoxia and ischemia may be unavoidable in some clinical settings such as renal transplantation. Further, inflammation and oxidative damage are closely linked in ischemia/reperfusion (I/R) injury and AKI [14], as ROS can promote immune responses and vice versa. Experimental models of AKI show that endothelium activation that promotes leukocyte recruitment and microvascular congestion, as well as altered nitric oxide (NO^•^) biosynthesis, mitochondrial dysfunction, and redox active iron, contribute to heightened ROS generation and oxidative damage.

### 2.1. Sources of ROS in Various Causes of AKI

Various patho/physiological ROS, including the free radicals superoxide anion (O_2_^•−^) and hydroxyl radical (OH^•^), and the non-radical oxidants hydrogen peroxide (H_2_O_2_), hypochlorous acid (HOCl), and peroxynitrite (ONOO^−^), may be relevant oxidants in AKI [5,15,16]. O_2_^•−^ is a significant precursor of ROS such as H_2_O_2_, HOCl and OH^•^, and can react with other radicals including NO^•^ to form reactive nitrogen species (RNS) such as peroxynitrite. Cellular O_2_^•−^ is produced by dysfunctional mitochondria in hypoxia, ischemia, and toxicity [3], and enzymically by plasma membrane and phagocyte NADPH oxidase (NOX). Limiting substrate or cofactors in injury or pathological conditions can uncouple nitric oxide synthase (NOS) to also generate O_2_^•−^ [15]. ROS, including those derived from O_2_^•−^, can induce lipid and protein oxidation observed in renal injury.

A causal role for ROS in ischemia-induced AKI was suggested in early studies in animal models showing significantly increased lipid peroxidation in kidney tissue after renal ischemia, which correlates with injury and tubular dysfunction [9]. Exploring this correlation further, several agents that inhibited ROS formation in vitro, including small molecular weight and enzymatic antioxidants and metal chelators, were effective in alleviating ischemic AKI [9,17]. Lipid peroxidation and DNA damage in ischemia are associated with the formation of 3-nitrotyrosine, a biomarker for ROS/RNS, suggesting that NO^•^, O_2_^•−^, and/or peroxynitrite, contribute to renal oxidative damage [18,19].

ROS may also be a causative factor in sepsis-mediated AKI. The extensive immune response induces severe renal vasoconstriction, kidney endothelial cell injury, and localized tissue hypoxia that supports ROS formation. Inflammatory cytokines and ischemia also activate vascular endothelium, recruiting immune cells that produce O_2_^•−^ via NOX, and HOCl from H_2_O_2_, and phagocyte myeloperoxidase (MPO) [16]. Inflammation-induced xanthine oxidase (XO) may also produce O_2_^•−^ [3]. Decreased plasma antioxidants (vitamins C and E, and thiols), and increased lipid peroxidation, are also found together with alterations in redox regulatory genes, such as mitochondrial superoxide dismutase (SOD), NOX, endothelial nitric oxide synthase (eNOS), heme oxygenase-1 (HO-1), and tumour necrosis factor (TNF) [16]. Mitochondrial dysfunction and bio-energetic failure is also evident in sepsis and mitochondrial complex I activity that correlates with reduced glutathione (GSH) and ATP levels, and is inversely associated with shock severity in non-surviving patients [20]. Further, inducible NOS (iNOS) is up-regulated in various organs, and shock severity is associated with NO^•^ levels [20]. Enhanced vascular NO^•^ production can outcompete SOD for O_2_^•−^, thereby promoting peroxynitrite and other ROS/RNS formation, and this may impact on vascular tone [21].

Rhabdomyolysis (RM) is a major cause of AKI in traumatic injury and severe burns. Extensive muscle myolysis releases large quantities of heme-containing myoglobin (Mb) resulting in myoglobinuria, severe renal vasoconstriction, and vascular dysfunction from obstruction by Mb-protein casts or uric acid crystals in kidney tubules [8]. Studies in animal models show that myoglobinuric damage is associated with lipid peroxidation and GSH depletion [10]. Redox active iron released from Mb may induce OH^•^ generation through degradation of low molecular weight peroxides. However, autoxidation of Mb, that is pH dependent and favored in acidosis, or oxidation of Mb by endogenous peroxides, can also generate protein-based radicals and ferric/ferryl heme, to promote radical-mediated reactions such as lipid peroxidation [22,23]. Potent vasoactive signaling molecules, e.g., isoprostanes, are found in animal models of RM, suggestive of lipid peroxidation in situ [24,25].

Nephrotoxicity accounts for a large cohort of AKI, as renal detoxification and/or filtration of various drugs exposes the kidney to a high toxin risk. AKI induced by common pharmaceuticals and radio-contrast dyes is a significant clinical problem [26]. Renal accumulation of drugs and/or metabolites can cause direct toxic effects on tubular cells, as well as microvascular inflammation and ischemia promoting ROS. Further, drug biotransformation in the kidney is performed by ROS-inducing renal enzymes, such as cytochrome P450 [10]. Antibiotics (gentamycin) and cancer therapies (cisplatin and cyclosporine A) induce kidney tissue lipid peroxidation and renal dysfunction via increased ROS formation, and iron release from renal cortical mitochondria in vitro and in vivo [10]. Drug-induced oxidative stress may also involve depletion of antioxidants, particularly the enzymic cofactor GSH, permitting unregulated ROS/RNS accumulation and renal cell injury [10,27].

### 2.2. Is Oxidative Stress Causally Related to Renal Dysfunction?

Outcomes from the influential study PICARD (Project to Improve Care in Acute Renal Disease), demonstrated that acute renal failure in critically ill patients was associated with significantly more oxidative stress than that observed in subjects without AKI, healthy controls, or end-stage kidney disease [11]. Thus, plasma protein thiols, employed as a surrogate measure of antioxidant capacity, were significantly decreased, and advanced protein oxidation products were significantly increased, in subjects with clinical AKI. Also, impaired renal function associated with increased plasma pro-inflammatory cytokines (IL-6, IL-8 and TNF-α), and further, cytokines and thiols were inversely related, suggesting that inflammation promotes oxidative stress in AKI. Oxidative stress may also be important in developing chronic kidney disease, as lipid peroxidation products associate with advancing disease, as does endothelial dysfunction and loss of plasma SOD, glutathione peroxidase (GPx) activity, and selenium [28].

While pre-clinical and human studies of AKI and renal insufficiency consistently associate oxidative stress/damage and renal dysfunction, there is limited direct demonstration of the role of ROS, so it is difficult to assign the latter a definitive casual role. Some recent studies have tried to address this using non-invasive in vivo imaging techniques, or more specific indicators, to track ROS formation in AKI. For example, a stable, electron paramagnetic resonance (EPR) spin probe injected into rats showed only partial recovery of kidney reducing (antioxidant) activity after renal I/R, despite improvements in renal function and tissue phospholipid oxidation, suggesting that ongoing oxidative stress depletes antioxidant reserves in renal ischemia [29].

Loss of ATP, and alterations in mitochondrial structure, are early events in AKI that contribute to bio-energetic dysfunction [3,10]. A recent study of endogenous and exogenous multi-photon imaging in vivo, assessed kidney mitochondrial redox state, structure, and function in rodents during ischemic and nephrotoxic AKI [30]. Alterations in mitochondrial NADH and proton motive force, as well as increased mitochondrial O_2_^•−^ levels in proximal tubules and fragmented mitochondria were observed, suggesting that this organelle is a major source of ROS, and that mitochondrial dysfunction is an important early event in renal ischemia [30]. In comparison, abnormalities in renal epithelial lysosomes and brush border cells after gentamycin treatment, preceded heterogeneous and sporadic alterations in mitochondrial morphology, NADH, and proton motive force, suggesting that mitochondrial dysfunction is a relatively late event in nephrotoxic AKI [30]. This study not only provided direct visualization of ROS alongside cell damage, but also highlighted the variation in pathophysiology and roles of ROS and mitochondrial dysfunction in different causes of AKI.

Unlike pre-clinical studies, direct measurement of ROS in renal injury/disease patients is practically limited to non-invasive biomarkers. Specific and stable markers of in vivo free radical mediated-lipid oxidation, such as isoprostanes, have been utilized to show substantial lipid peroxidation in patients with RM [31], and with progression of chronic kidney disease [32]. Increased plasma F_2_-isoprostanes are also found in renal failure in sepsis [33], and with postoperative AKI [34]. In the latter study, hemoprotein-induced oxidative damage was suggested to play a role in the pathogenesis of AKI. Isofurans contain a substituted tetrahydrofuran ring and are also derived from free radical-mediated lipid oxidation, but are favoured with high oxygen tension as can occur in mitochondrial dysfunction [35]. Plasma isofuran levels similarly increase in sepsis [33], and cardiopulmonary bypass [34] patients with AKI, and in chronic kidney disease [35].

## 3. Mitigation of Renal Oxidative Stress and Therapeutic Benefit

Whilst ROS perform important roles in cell signaling and physiological processes [36], they are clearly linked with acute and chronic renal injury. Antioxidants participate in ROS detoxification and decomposition processes to maintain redox balance in vivo, and to protect against adverse oxidation. The major endogenous antioxidants mitigate source ROS, such as O_2_^•−^ and H_2_O_2_, and their reaction with other radicals [37], and can also directly interact with pertinent non-radical oxidants derived from O_2_^•−^, such as HOCl and peroxynitrite [38]. Notable antioxidants include the vitamins C (ascorbate) and E (tocopherol family), GSH, antioxidant proteins such as SOD, catalase and GPx, and proteins that sequester metals (ferritin, metallothionein) or degrade heme (HO-1) (Nath, 2014). Exogenous and synthetic compounds may act as direct antioxidants, or may activate adaptive systems such as the nuclear factor E2-related factor (Nrf2) signaling pathway that regulates endogenous antioxidant enzymes and cytoprotective genes [39]. Antioxidants that quench ROS or boost the endogenous antioxidant pool, may be therapeutic in AKI.

### 3.1. Inhibition of ROS Source

A wide range of free radical scavengers, metal chelators (that inhibit redox cycling of bound metals), and inhibitors of ROS enzyme sources, decrease lipid peroxidation, DNA damage, and/or protein oxidation/nitration, and this is closely associated with improved renal function and inflammation in animal models of AKI, and related renal cell studies. These outcomes have prompted attempts to remove potential ROS sources with inhibitors, genetic knockout techniques, or antioxidants, to not only assign a mechanistic role for ROS in AKI, but also identify ROS as a target for therapeutic advantage.

#### 3.1.1. Pro-Oxidant Metals

The role of pro-oxidant metal ions and metal-containing heme has been intensely studied in AKI, as potent ROS such as OH^•^ and oxidised lipids, that are vasoactive, can be formed by redox active metals in the presence of O_2_^•−^/peroxide, and because the latter are increased in injury. Several studies affirm that metal chelators, such as desferrioxamine, are protective against oxidative damage, and renal dysfunction in animal models of nephrotoxic and injurious AKI [40,41,42]. These studies also show renal protection and reduced lipid oxidation afforded by so-called OH^•^ scavengers, and more specific antioxidants such as SOD, GSH, and vitamin E [10].

One source of released iron is heme-containing proteins in mitochondria or endoplasmic reticulum or Mb, the latter being released in large amounts in RM-induced AKI [10]. Other hemoproteins may also contribute to oxidative stress. For example, cytochrome P450 inhibitors modulate kidney iron levels and improve renal function and injury in both RM-mediated AKI and cisplatin nephrotoxicity in rats, suggesting that redox cycling of this enzyme is important to renal injury [43,44]. Down-regulation of cytochrome P450 2E1, using a specific transcription inhibitor, also modulates lipid peroxidation, and associates with normalising antioxidant enzymes and iron levels, and is reno-protective in RM-induced AKI in rats [45].

Maintaining Mb heme-iron in a chemically reduced (Fe^2+^) state also appears to ameliorate AKI and renal dysfunction, and may explain the positive effects of endogenous antioxidant replacement, and also some of the action of desferrioxamine that can reduce Mb heme [23]. Further, inhibition of Mb heme redox cycling by alkalinisation [24], or inhibition of endogenous lipid peroxidation with acetaminophen, prevents isoprostane formation and renal injury associated with RM [25]. Notably, acetaminophen was effective in reducing oxidant injury and renal dysfunction when administered either pre- or post-treatment. Whether acetaminophen removes seed lipid peroxides, or also acts on renal inflammation, is not known. In any case, removing pro-oxidant forms of iron by antioxidant therapy or chelation appears to be efficacious, particularly in injurious AKI. 

#### 3.1.2. Superoxide Radical Anion and Derived ROS Sources (NOX, Mitochondrial ROS)

The available evidence indicates that SOD and other antioxidant enzymes are decreased in pre-clinical models of AKI [46], and genetic impairment of SOD increases sensitivity to AKI in ischemia [47] and chronic hypoxia [48]. Generation of O_2_^•−^, oxidative damage, and reduced SOD and catalase activity, persist following transient renal ischemia in rodents, and associate with functional defects that promote kidney disease. Long-term treatment with a SOD mimetic (MnTMPyp) [49] or the NOX inhibitor apocyanin (which can also act as an antioxidant radical scavenger [50], alleviated oxidation parameters and reduced the functional defects in this injury model [3]. Notably, NOX gene levels did not appear to be altered, however, other pro-oxidant genes were increased, including MPO and dual oxidase I (shares homology with NOX), whereas extracellular GPx3 was chronically decreased [3]. In comparison, NOX2/4 mRNA and protein are elevated in a pre-clinical model of nephrotoxicity [51], and both total NOX activity and NOX4 protein are increased in contrast dye-induced (CI-)AKI in hypercholesterolemic rats [52]. Also, polymorphisms affecting activity in NOX p22phox subunit gene associate with oxidation biomarkers and adverse outcomes in acute renal failure patients [53].

Blockage of ROS production from O_2_^•−^ sources such as XO, NOX, and mitochondria, alleviates animal model AKI. Allopurinol, a XO inhibitor, modulates oxidative damage and improves renal function in renal ischemia [54] and RM-induced AKI [55]. Allopurinol also reduces vascular oxidative stress and improves endothelium function in chronic kidney disease [56,57]. Apocynin, a prototypical inhibitor of NOX, is protective against renal dysfunction and lipid peroxidation [3], and loss of SOD after I/R in rats with a similar efficacy to allopurinol [58], although whether protection is due to direct inhibition of NOX or the inhibitor antioxidant activity, per se, is not clear. Treatment with a combination of apocynanin and allopurinol failed to show any further efficacy than individual drug administration [58], suggesting that a common target, i.e., ROS, was adequately quenched by either inhibitor. In another recent study in rats, apocynin normalized kidney MPO and GPx protein, reduced lipid peroxidation, and improved kidney function after renal ischemia [59].

Mitochondrial structural damage is an early, distinctive marker in AKI, and is linked to increased production of ROS and activation of cell death pathways, and an inflammatory response that potentiates ROS formation [3]. Whether mitochondrial ROS are causative or formed subsequently in AKI is not known, however, there is ongoing interest in the development of therapeutic antioxidants specifically targeted to this organelle, and several show efficacy in preclinical AKI, and are the focus of clinical trials, especially for I/R injury [60]. For example, the ubiquinone analogue MitoQ effectively protects against kidney dysfunction and oxidative damage in renal I/R [61], nephrotoxic AKI [51], and cold storage ex vivo indices of oxidative stress and kidney damage [60]. Also, mitochondrial targeted peptides thought to protect cardiolipin from cytochrome C peroxidation, show efficacy against oxidative stress, tubular cell damage, and dysfunction in renal I/R injury [60].

#### 3.1.3. NO^•^ Derived ROS/RNS

Peroxynitrite causes oxidation and/or nitration of lipid and protein, amino acids and DNA, depletion of thiols and antioxidants, and oxidation of heme proteins. Nitration of tyrosine residues is often used as a biological marker of peroxynitrite generation, and 3-nitrotyrosine is found in ischemic, nephrotoxic and injurious AKI. However, it should be noted that several peroxidases, including MPO, provide an alternative mechanism of protein tyrosine nitration via NO^•^ oxidase activity [62]. Thus, in addition to SOD, MPO may be considered a modulator of NO^•^ signaling during inflammation [62], and this may be relevant in sepsis where the inflammation response may contribute substantial MPO. Thus, considering that iNOS and MPO are up-regulated in infection/inflammation, the observation of 3-nitrotyrosine in vivo in various causes of AKI, is probably restricted to a nonspecific indication of ROS/RNS.

An imbalance in NO^•^ and O_2_^•−^ production during hypoxia and I/R injury may contribute to renal cell damage [18,19]. However, use of agents to globally inhibit NO^•^ production, including that from constitutive eNOS, is not reno-protective in I/R injury [63]. Interestingly, iNOS is constitutively expressed in the kidney [64], emphasizing a role for NO^•^ in normal renal function [15]. However, sustained NO^•^ release from iNOS may also be pathogenic, as mice deficient in iNOS are resistant to renal I/R injury [65]. Moreover, specific inhibition of iNOS reduces oxidative and nitrosative damage, and renal dysfunction in animal models of renal ischemia [18,66], sepsis [67,68], and nephrotoxic AKI [27].

### 3.2. Antioxidant Interventions; Supplementation and Up-Regulation

Several strategies for modulation of AKI using antioxidant compounds have been tested in human and animal studies [6,7,8]. These include increasing bioavailability by intervention with nutrient-derived and/or synthetic antioxidants, identifying new reno-protectants with antioxidant activity, and targeting of antioxidants to specific ROS cellular domains (e.g., mitochondria). Also, anti-inflammatory agents may potentially reduce ROS via stabilising endothelium function and NO^•^ bioactivity, as well as up-regulating gene responses linked to antioxidation and cytoprotection.

#### 3.2.1. Small Molecular Weight Endogenous/Nutrient or Synthetic Antioxidants

Several small molecular weight compounds with antioxidant and ROS scavenging actions improve renal function and decrease tubular damage. For example, edaravone (3-methyl-1-phenyl-2-pyrazolin-5-one; norphenazone, MCI-186) shows efficacy in ischemia and is an approved treatment for stroke in Japan [69]. It has been widely reported to inhibit oxidative damage and lipid peroxidation in ischemia, however, it also shows anti-inflammatory properties that may be unrelated to its antioxidant activity [69]. Edaravone attenuates ROS radical generation in kidney tubular cells in vitro, and lipid peroxidation measured as aldehyde-modified proteins in vivo, and ameliorates renal dysfunction in I/R [70] and nephrotoxicity [71] in rats. Edaravone also improves survival rates in warm and cold I/R injury in rats [72] and dogs [73], and in the latter, significantly improved renal function and reduced renal tubular cell damage, lipid, and DNA oxidation [73], suggesting that it may prevent preservation injury in transplantation. Despite these positive effects, there are reports of edaravone treatment causally associated with AKI in ischemic stroke, however, this has not been validated by a recent survey [74].

*N*-acetylcysteine (NAc) is a synthetic derivative of cysteine and precursor of GSH, and exhibits ROS scavenging activity via its sulfhydryl group. It is protective in ischemic, nephrotoxic, and RM-induced AKI in animal models [8], and improves kidney function, renal GSH and systemic oxidative stress, and reduces renal inflammation. However, it has no effect on urinary isoprostanes, suggesting cellular activity in addition, or unrelated, to a primary antioxidation mechanism. NAc has been tested in several clinical studies of CI-AKI [75]. However, on balance, NAc shows no overall benefit in preventing or treating CI-AKI in humans, and meta-analysis of these trials highlight heterogeneity, under-reporting of negative/no benefit, and confounding serum creatinine levels as possible contributors to the neutral effect. In addition, inadequate animal models for CI-AKI may have hampered translation. Similar to CI-AKI, pre-, intra-, or post-operative use of NAc in clinical trials to preserve renal function in cardiac or abdominal aortic surgery has largely failed to show benefit [7].

Endogenous or dietary antioxidants are protective against oxidation and/or inflammation and kidney damage in AKI. For example, vitamin E and selenium (that can enhance activity of GSH-dependent antioxidant enzymes) attenuate nephrotoxicity [7]. Interestingly, Se supplementation inhibited renal oxidative damage and inflammation, yet was not reno-protective in an animal model of RM-mediated AKI [76]. Vitamin C also attenuates oxidative damage, inflammation and renal injury in several animal models, including CI-AKI [75], and other nephrotoxic AKI [7], ischemia- [5,6] and RM-induced renal injury [8,77] (and see Table 1 for results of recent vitamin C intervention studies on oxidative damage and/or antioxidant status and kidney function in animals). Loss of GSH or GSH reductase activity worsens renal function in RM [10] and renal ischemia [78]. Conversely, supplementation of GSH decreases renal cell/tubule oxidative injury [78,79] and improves renal function in AKI [80]. NAc, that can increase intracellular GSH, and the GPx mimetic ebselen, both show efficacy in AKI in animal models [3,5]. Ebselen may also be protective by scavenging peroxynitrite [5] thereby inhibiting protein modification by this potent oxidant.

Endogenous antioxidants act in coordinated networks to mitigate oxidative damage, and this may help explain why they are efficacious in AKI. The low-molecular weight antioxidants, α-tocopherol and ascorbate, inhibit propagation reactions, and are effective terminating antioxidants. However, they also act as co-antioxidants to spare other antioxidants and transfer radicals away from susceptible moieties [90]. GSH performs multiple ROS detoxification roles, including ROS scavenging, preventing protein thiol oxidation, as a co-factor for the GPx enzyme family that reduces peroxides and detoxifies xenobiotics via glutathione S-transferase conjugation. GSH is regenerated from its oxidation product, GSSG, by glutathione reductase and cofactor NADPH. Further, mutual maintenance of ascorbate and GSH may occur in vivo, as ascorbate can maintain intracellular GSH, GSH can overcome scurvy, and vitamin C is recycled via GSH and/or GSH or NADH-dependent enzymes [91].

In addition to endogenous antioxidants, several dietary plant polyphenols and flavonoids including curcumin, quercetin, resveratrol, and red wine polyphenols, appear to be efficacious in RM- [7,9] and ischemic AKI [6] in animal models. While several of these phytochemicals display antioxidant activity in vitro, they are well known to activate the Nrf2 signaling cascade [39] that up-regulates several antioxidant genes, including enzymes that interconnect H_2_O_2_ and thiol modification (e.g., GSH biosynthesis and GSH-dependent enzymes, thioredoxin, peroxiredoxin, and GPx). Nrf2 also activates transcription of HO-1, and ferritin that can mitigate AKI and renal injury [5,7]. Synthetic phenols with antioxidant activity may also act via Nrf2 to up-regulate reno-protective HO-1 (see below and [77,92]).

#### 3.2.2. Antioxidant Enzymes

Enhancement of antioxidant enzyme activity appears to be protective in several animal models of AKI. Early studies of ischemia showed that SOD or catalase administration attenuates ROS in proximal tubule injury after hypoxia in vitro [93], and that SOD diminishes oxygen radicals in vivo after renal ischemia in rabbits [94]. Also, SOD improved renal function and reduced kidney tissue injury and cortical mitochondrial lipid peroxidation in rats [9]. Further studies confirmed that SOD reduced ROS and was cytoprotective to renal cells in vitro and in vivo (reviewed in [3,10]). Pharmacologic agents with SOD mimetic activity (Tempol, MnTMPyP) attenuate sepsis- [95,96] and ischemia-induced AKI [97,98]. Further, MnTMPyP attenuates chronic increases in ROS and oxidative damage, and a reduction in SOD associated with kidney fibrosis after ischemic AKI [49]. In animal sepsis, MnTMPyP blocked O_2_^•−^ and peroxynitrite formation, and reversed functional kidney deficits when added 6 h post-septic insult, suggesting that antioxidant intervention is beneficial, and that halting ROS formation can ameliorate microvascular failure and renal injury [96].

Over-expression of MnSOD, but not catalase, attenuates cisplatin-induced renal epithelial cell injury in vitro [99], further suggesting that O_2_^•−^ is important in AKI. Also, hyperglycemia that contributes to diabetic nephropathy, induces O_2_^•−^ within mitochondria and inactivates complex III, and these changes can be alleviated by MnSOD over-expression [100]. MnSOD efficiently converts O_2_^•−^ to H_2_O_2_, allowing ROS to exit the organelle. However, renal MnSOD inactivation (up to 50%) associated with increased mitochondrial O_2_^•−^, has been demonstrated in mouse sepsis, and this can be attenuated with the mitochondria-targeted antioxidant Mito-TEMPO [101]. Further, Mito-TEMPO mitigated renal mitochondrial and circulation dysfunction, together with doubling the survival rate, and was effective when administered post-septic insult. Whether other low-molecular weight cyclic nitroxide SOD mimetics, that also show anti-inflammatory activity independent of radical quenching [102], can provide reno-protection, requires further investigation. Similarly, the SOD mimetic, Mito-CP, also targets mitochondria and protects against tubular cell dysfunction, injury, apoptosis, and inflammation in mice administered cisplatin, accompanied by reduced NOX2/4 mRNA and protein, lipid oxidation, protein nitration, and pro-inflammation markers (MPO, ICAM-1) [51]. Thus, targeting the initial toxic insult that induces mitochondrial ROS with antioxidants may prevent further ROS formation facilitated by inflammatory cell infiltration and NOX [51].

#### 3.2.3. HO-1 and Heme Metabolism

Heme oxygenase, normally found in the reticulo-endothelial system, can be rapidly induced in various tissues as a stress (including oxidative) protein, including the kidney. It is considered an antioxidant as it metabolizes heme from various proteins, including Mb, allowing clearance and sequestration of redox-active iron (by ferritin), and its re-utilization. In addition, heme metabolism by HO-1 produces biliverdin that can be converted to the plasma antioxidant bilirubin and CO; the latter participates in cell signaling and is cytoprotective in the vasculature [103]. Both HO-1 and ferritin are induced as an adaptive response to myoglobinuria in rats injected with glycerol to induce RM, and treatment with a competitive HO-1 inhibitor worsens renal function, while HO-1 induction by hemoglobin is protective [104]. However, it is well known that exposure of cells to heme renders them sensitive to ROS, such as H_2_O_2_ [105]. This suggests a fine balance between adaptive and maladaptive responses to heme, where small pre-treatment doses may be protective, similar to ischemic pre-conditioning (see below), and reliant on cell signaling processes involving antioxidant, anti-inflammatory, and vascular cytoprotective pathways.

Pharmacologic and genetic manipulation of HO-1 in animal studies suggests HO-1 is protective in other causes of AKI, including nephrotoxicity, ischemia, and sepsis [7,103]. For example, inhibition of HO-1 hinders recovery of renal function in rats after renal ischemia [106]. Transgenic deficiency in HO-1 renders mice more susceptible to renal failure and injury after cisplatin treatment and, hemin addition to renal proximal tubule cells in vitro induces HO-1 and a pronounced cytoprotective effect [107]. Furthermore, HO-1 is protective in AKI following renal transplantation, and its products inhibit tubulo-glomerular feedback and thrombotic microangiopathy in sepsis [103]. Moreover, loss of proximal tubule ferritin worsens AKI [108], and HO-1 knockout mice display increased lipid and protein oxidation, and iron deposition in kidneys [109]. HO-1 also confers protective effects in specific organelles, such as mitochondria, and appears to be induced in specific renal sites aligned with the AKI insult, and targeting of HO-1 to the proximal tubule is protective in nephrotoxicity [110].

#### 3.2.4. Maintenance of Endothelial Function

Biomarkers of endothelium dysfunction are associated with increased risk of AKI in critically ill patients, suggesting that endothelial cell activation predisposes to developing kidney injury [111]. Ischemia can drive endothelium activation by inducing chemo/cytokines that recruit immune cells and allow their transmigration, and maintaining endothelium function may be important in limiting I/R injury in AKI. For example, ICAM-1 induces leukocyte adhesion to endothelial cells, and up-regulation of inflammatory mediators causing endothelium dysfunction and administration of an ICAM-1 antibody, genetic knockout of ICAM-1, or prevention of neutrophil infiltration in mice attenuates renal ischemia-induced AKI [112]. Various anti-inflammatory agents that hinder phagocyte infiltration, NF-Kb, and fibrosis mediators, are also effective in preserving renal function in various AKI [6,8]. I/R can induce direct endothelial cell damage via ROS and/or mitochondrial dysfunction, thereby interfering with NO^•^ homeostasis and vascular function. Several positive modulators of NO^•^ via eNOS, and selective inhibition of iNOS and/or peroxynitrite formation via SOD mimetics, reverse renal dysfunction and oxidative injury in various AKI models [5,6,8].

Pre-conditioning by imposing a stress prior to subsequent injury may be effective in renal ischemic injury. As ROS are important signaling molecules, short bursts of ischemia can promote signaling cascades that protect renal cells from more prolonged I/R injury. Thus, transcription activators such as Nrf2 and hypoxia-inducible factors (HIF) up-regulate stress-response and cytoprotective genes, e.g., HO-1, and these may be integral to the protective effects observed in remote and pharmacological pre-conditioning strategies [3]. For example, pre-conditional induction of HIF protects against ischemic AKI in rodents [113]. Some antioxidants (ascorbate, SOD) can block protection afforded by ischemic pre-conditioning in the heart [114], further indicating that ROS are important signaling molecules in vivo. A role for up-regulation of renal NO^•^ production and improved vascular function via enhanced NO^•^ bioavailability has also been suggested, as pre-conditioning benefits are reduced in NOS-inhibited or eNOS deficient mice [115].

## 4. Vitamin C and Renal Protection

**Figure 2:**
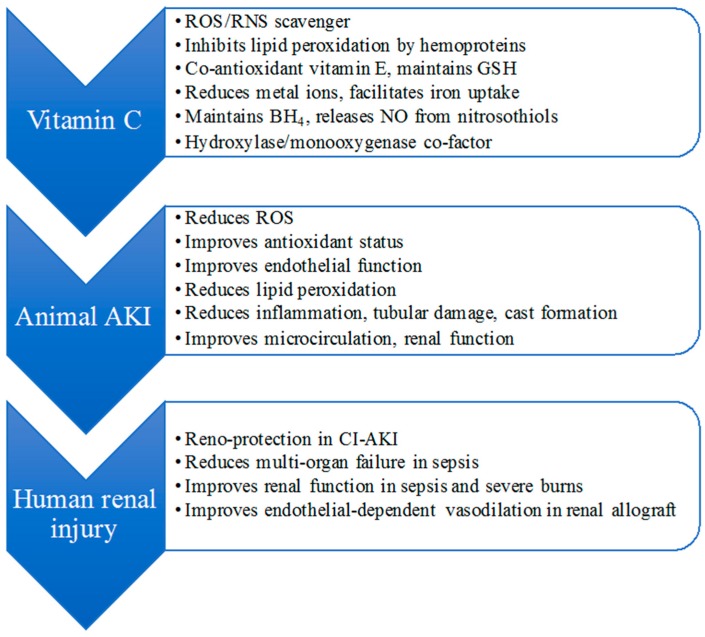
Key activities of vitamin C and proposed benefit mechanisms in acute renal injury. BH4 = tetrahydrobiopterin; RNS = reactive nitrogen species; CI-AKI = chemical induced acute kidney injury

There is substantial interest in vitamin C (ascorbate) as a therapeutic antioxidant in renal dysfunction, and vitamin C supplementation has been shown to be protective against ischemic, injurious and toxicity-induced oxidative stress, and kidney dysfunction/AKI in animal models, and human studies of critical illness (see Figure 2, overview of proposed mechanisms of vitamin C reno-protection). Ascorbate is an essential nutrient obtained from the diet, and is a highly effective non-protein reducing agent capable of donating electrons in various enzymatic and non-enzymatic reactions [116]. In this capacity, it can undergo two consecutive one-electron oxidations to yield first ascorbyl radical, and then dehydroascorbic acid (DHA), and both of these forms are recycled to ascorbate by thiols/GSH and/or GSH, or NAD(P)H-dependent enzymes, effectively enhancing the potential protective action of ascorbate.

Ascorbate acts as an enzyme cofactor in several hydroxylase reactions by maintaining active-site metals in a reduced (active) state. In this regard, it is essential for functional collagen synthesis, and vitamin C deficiency adversely affects wound healing and blood vessel wall integrity, and causes scurvy. It is also a cofactor for cytoplasmic prolyl hydroxylases that control activation of HIF and up-regulation of pro-survival glycolytic and angiogenic genes [117]. In addition to these activities, vitamin C is proposed to have an important physiological role as an effective in vivo antioxidant. The basis of this is related to its low reduction potential, that allows direct interaction with a wide range of physiological ROS/RNS [118], and a large body of in vitro evidence demonstrates the effectiveness of ascorbate in inhibiting biomolecule oxidation [116].

Ascorbate is an efficient ROS/RNS scavenger in both tissue and plasma, and these non-enzymatic, antioxidant bioactivities have prompted therapeutic investigations of ascorbate in AKI and renal injury. Thus, ascorbate protects against ROS damage to protein, lipid, DNA, and carbohydrate in aqueous milieu both extra- and intra-cellularly, and in several ROS-induced pathologies [116,117]. It scavenges radicals (O_2_^•−^, (hydro)peroxyl, nitroxide) and non-radical (HOCl, peroxynitrite) oxidants, and reduces levels of α-tocopheroxyl radical in lipids and membranes, allowing recycling of vitamin E, and inhibition of lipid peroxidation [90], and can spare GSH and protein thiols. It is also safe, with high pharmaco-economic benefit, is fast acting on systemic antioxidant status, and large quantities can be administered acutely with minimal adverse effects via various modes [117,119].

Intestinal uptake and renal re-absorption is important in ascorbate bioavailability, as humans, unlike most mammals, cannot synthesize the vitamin de novo. Circulation levels of ascorbate are tightly controlled in the micromolar range, whereas intracellular levels are much higher [120]. Ascorbate is distributed by vitamin C membrane transporters (SVCT) in nucleated cells, whereas DHA, the 2e-oxidation product of ascorbate, is transported by Na^+^-independent glucose transporters (GLUT), and is rapidly reduced intracellularly. Interestingly, activation of the HIF transcription factor during ischemia also increases expression of the GLUT-1 transporter [117], and this may be a mechanism to bolster ascorbate, as well as glucose, for energy metabolism. Ascorbate oxidation can substantially increase DHA levels allowing vitamin C accumulation in various cell types, and this may be important to its antioxidant function [120]. Thus, large amounts of ascorbate can be made available during an inflammation response, e.g., phagocytic cells undergoing respiratory burst, to balance ROS production. However, genetic polymorphisms in human vitamin C transport genes affect plasma ascorbate levels, and hence disease risk and individuals with low dietary intake may be more susceptible to the effects of genetic variation [121].

Whether ascorbate performs antioxidant roles in vivo is largely unproven. Its clinical use is restricted to prevention of scurvy and promoting intestinal non-heme iron absorption, though it is currently being investigated as a pro-drug in cancer [117]. Epidemiological studies consistently show that low plasma ascorbate levels are associated with increased chronic disease risk, though vitamin C supplementation is yet to show definitive benefits [122]. Low plasma vitamin C is a risk factor for mortality and adverse cardiovascular events in hemodialysis patients [123], and AKI co-morbidities, such as diabetes, are associated with vitamin C deficiency [124]. The renal system is important in vitamin C re-absorption [125], and impairment may affect plasma ascorbate levels. Patients with renal dysfunction, such as septic, critically ill, and elderly, demonstrate low ascorbate levels [119], and bolstering vitamin C intake may prevent ROS-mediated renal damage in AKI.

### 4.1. Evidence for Vitamin C Efficacy in Animal Models of AKI and Proposed Actions

#### 4.1.1. Vitamin C and Nephrotoxicity

Animal studies consistently show efficacy of vitamin C supplementation in nephrotoxic AKI by reducing ROS and inflammation damage [7]. This positive benefit on renal function is predominantly attributed to its antioxidant function and ability to reduce ROS arising from the initial toxic insult and/or secondary wave ROS induced by inflammation (Table 1). Vitamin C also appears to maintain GSH [126]. A large analysis of pre-clinical studies of aminoglycoside antibiotic-induced nephrotoxic AKI showed that both natural and synthetic compounds, including vitamin C with attributed antioxidant activity, are reno-protective [127]. Vitamin C also protects against NSAID-induced AKI in rats by improving kidney function and renal lesions, serum oxidative stress, and tissue inflammation, comparatively to vitamin E administration [89]. It is also protective against nickel-induced toxicity in mice, by improving renal function, inflammation and renal tubular degeneration, and necrosis [128]. This finding supports earlier evidence of decreased nickel-induced oxidative stress in other organ systems with ascorbate [129]. Interestingly, vitamin C supplementation reduces nickel accumulation in the kidney [128], suggesting benefit independent of ROS scavenging.

Ascorbate also improves RM-induced renal injury in animal models (Table 1). Thus, rats administered a bolus of vitamin C intraperitoneally immediately after RM induction, showed significant reductions in kidney tissue lipid peroxidation, increased antioxidant enzymes, and reduced tissue iron content and tubular necrosis [85]. Yet, no significant improvements to renal function were observed. This may be partially explained by the low dose of vitamin C chosen. However, studies in our lab have similarly demonstrated a lack of amelioration of AKI with antioxidant (synthetic polyphenol, vitamin E, selenium) supplementation in animal models of RM, despite ameliorating oxidative stress and decreasing biomarkers of inflammation, together suggesting that oxidative stress may not be causally related to renal dysfunction [76,130]. We recently compared treatment with vitamin C or the synthetic polyphenol tert-butyl-bisphenol (3,3′,5,5′-tetratert-butyl-biphenyl-4,4′-diol) in a murine model of RM-induced AKI [77]. Tert-butyl-bisphenol shows antioxidant activity similar to ascorbate, and inhibits Mb-induced renal cell dysfunction in vitro [131]. Both ascorbate and tert-butyl-bisphenol comparatively decreased plasma and kidney oxidative markers, inflammation and tissue kinase activity (Table 1) when administered alone or in combination [77]. However, only vitamin C showed potential clinical benefit and reduced proteinuria, plasma urate and renal tubule casts. This data suggests that antioxidants with enhanced water solubility, such as ascorbate, may prevent intratubule obstruction and tubular epithelial cell damage by Mb casts or urate crystals. Alternatively, ascorbate may exhibit protective activities adjunct to its ROS scavenging/antioxidant activity [77]. Vitamin C can positively affect endothelium function and exert anti-inflammatory actions, and anti-inflammation and vasoprotective therapies attenuate RM-induced AKI [8].

In addition to the above, ascorbate displays a multifunctional antioxidant role in animal models of cell-free hemoglobin exchange to prevent heme protein-mediated oxidative stress in vivo [132]. Thus, EPR spectroscopic studies show that ascorbate scavenges globin-centered radicals and reduces plasma methemoglobin (metHb, Fe^3+^) and ferryl hemoglobin (Fe^4+^-oxo), to remove the potential for ROS formation from peroxide/redox active heme-peroxidase reactions. Erythrocytes promote reduction of metHb by rapid recycling of ascorbate from ascorbyl radical. These antioxidant actions of ascorbate in plasma and whole blood may be relevant in reducing kidney damage when large amounts of heme proteins are released into extracellular spaces, such as in trauma and RM-induced AKI [132].

#### 4.1.2. Vitamin C and I/R-Induced AKI

Vitamin C supplementation is also associated with improvements in I/R-induced AKI, again, largely associated with ROS scavenging and improved antioxidant status. Thus, vitamin C administration improved plasma levels of antioxidant enzymes in a model of canine renal allograft [133,134], and reduced renal lipid oxidation and reversed loss of GSH in rat renal I/R [81]. The latter study demonstrated cytoprotective and antioxidant efficacy within a short period of I/R, and with one bolus dose of vitamin C pre-ischemia, suggesting that it may be beneficial and practical in defined elective procedures, such as renal transplantation. A renal ischemic injury study in mice also showed improved kidney function and decreased tubule cell injury with vitamin C pretreatment that was associated with decreased renal lipid oxidation and improved SOD and GSH levels [84]. In this study, vitamin C also significantly improved kidney NO^•^ levels and in vivo arterial resistance and vascular reactivity of excised renal arteries, indicating that ascorbate protects vascular function by direct ROS scavenging, and/or via up-regulation of SOD, to prevent renal injury.

Ascorbate is an electron donor for peptide alpha-amidating monooxygenases responsible for steroid and peptide hormone stability and activity. It is involved in progesterone biosynthesis [135], and progesterone shows similar antioxidant and anti-inflammatory activities to ascorbate in several diseases, including I/R-induced AKI. For example, progesterone mitigates oxidative stress and inflammation, and up-regulates antioxidant enzymes, and improves renal function in an animal model of renal I/R [87]. Interestingly, antagonism of progesterone receptors in male rats exposed to renal I/R abolished the antioxidant and anti-inflammation effects of vitamin C, suggesting the involvement of steroid receptors in ascorbate-mediated reno-protection [87].

Acute I/R injury to renal tissue from remote organ damage/surgery can be alleviated by vitamin C. Thus, renal ischemia injury in rats induced by abdominal aortic surgery that increased plasma and tissue lipid oxidation and acute inflammation, was attenuated with vitamin C, similarly, or more effectively, than a synthetic prostaglandin (PGI_2_) analogue (Iloprost) [88]. PGI_2_ inhibits platelet activation and is an effective vasodilator, and Iloprost is used clinically for pulmonary hypertension and ischemia. Thus, vitamin C’s ability to inhibit lipid peroxidation and reduce inflammation, may prevent platelet aggregation and leukocyte adhesion [88]. Indeed, an earlier study showed that vitamin C decreases venous blood platelet activating factor (PAF) and PAF-like lipids during reperfusion after renal I/R in rabbits and rats [136]. PAF is a potent phospholipid activator of vascular and immune responses, and is up-regulated in pathological conditions, and some lipid oxidation products have PAF-like activity. The decrease in PAF activity was associated with decreased inflammation (specifically MPO activity) and DNA oxidation, and amelioration of kidney dysfunction and tubulointerstitial damage, suggesting that ascorbate can intervene in the oxidative-inflammatory response in I/R. In another study, vitamin C reduced lipid oxidation, inflammation and kidney injury, and partially improved renal oxygen delivery and consumption [83]. Despite these positive effects, vitamin C had no effect on kidney hemodynamics and urine output, reminiscent of other studies where antioxidants improve renal oxidative stress, damage or inflammation but do not improve kidney function [77].

#### 4.1.3. Positive Effects of Vitamin C on Endothelial Function and Vascular Tone in Renal Injury

Ascorbate is vaso-protective of endothelium function, and this may be important in renal injury. Several mechanisms have been proposed, including enhancing NO^•^ bioavailability by up-regulating eNOS, and/or increasing its activity independently of, or via, maintaining tetrahydrobiopterin (BH_4_) [137]. Ascorbate may also maintain vessel integrity via scavenging ROS/antioxidant activity, preventing injury and/or inflammation, or via its other known physiological role as a co-factor of hydroxylase enzymes important in vascular structure/function [120]. Some animal AKI studies have compared the effect of l-arginine (NO donor) and vitamin C supplementation on biomarkers of lipid, DNA and protein oxidation, and kidney function, and have demonstrated superior protection afforded by vitamin C [86,138]. These improvements in renal function and oxidative stress markers in I/R-induced AKI in rats, may involve NO/soluble guanylyl cyclase (cGC), as inhibitors of this pathway (L-NAME and methylene blue) reduced the reno-protective effects of vitamin C [82]. Further, ascorbate increased tissue GSH and nitrate/nitrite levels, suggesting a preservation of NO^•^ levels. Chemical NO^•^ donors similarly reduce renal I/R in animal studies [6]. 

In addition to ischemia, vitamin C shows benefit in animal models of sepsis by improving edema, vascular tone, blood flow and pressure, platelet adhesion, coagulation, and survival [139]. The proposed mechanisms include decreased ROS/RNS, NOX, iNOS, and improved pro-inflammatory markers and GSH [119]. Vitamin C may be therapeutic in sepsis via NO^•^ maintenance, as alleviation of septic symptoms and improved capillary blood flow observed with ascorbate injection or BH_4_ superfusion is not evident in eNOS knockout mice [139]. Ascorbate can also stimulate eNOS activity in experimental sepsis via modulation of phosphorylation status, whereas other antioxidants such as NAc and trolox do not exhibit this activity [140]. Additionally, ascorbate may prevent endothelial barrier dysfunction in sepsis by modulating NOX derived ROS and peroxynitrite generation, thereby protecting the distribution of the endothelial tight junction protein occludin [140].

It is noteworthy that most mammals synthesise vitamin C de novo, and therefore, the overwhelming majority of vitamin C intervention studies that show benefit in animal AKI are performed on species (rats and mice) that are not deficient. This may suggest that endogenous levels of vitamin C are compromised in severe ischemic, nephrotoxic and/or injurious AKI, and that renal reabsorption is important in maintaining systemic vitamin C. Alternatively, vitamin C biosynthesis, that depends on adequate nutrient supply and liver function, may also be perturbed in these injury models.

### 4.2. Antioxidant Therapy in Human Renal Injury

AKI causes a high incidence of morbidity and mortality. Preventing AKI largely involves attempts to mitigate the inducing drug/injury/illness and renal replacement therapy (dialysis) to remove fluid overload and uremia, balance electrolytes, and correct metabolic acidosis. Addressing imbalances in nutrient-derived antioxidants, such as vitamin C, particularly after traumatic injury and in critically ill and elderly patients that show depletion of plasma antioxidants, may prevent renal injury [119,140].

Despite data showing positive benefit of antioxidants in animal models of AKI and renal injury, translation of antioxidant therapy to human studies has been of limited success. Thus, Nac has undergone several trials, but has proved largely inconclusive in alleviating CI- and other AKI [75] or chronic kidney disease [5]. It does however show some benefit in end stage renal disease and kidney transplantation. Vitamin E shows contrasting effects, either reducing chronic kidney disease risk, or displaying no benefit [5]. A clinical trial of the Nrf2 pathway enhancer bardoxolone methyl on end stage renal disease among type 2 diabetes patients and chronic kidney disease, was halted because of increased mortality (cardiovascular events) in the treatment arm [141].

#### 4.2.1. Reno-Protection in CI-AKI

In comparison to other antioxidants, vitamin C does appear to mitigate microvascular dysfunction and renal failure in I/R and sepsis. For example, vitamin C shows promising reno-protection in AKI [5,75]. Several controlled human studies have been now been performed with vitamin C supplementation prior, during, or post contrast dye procedures, usually coronary angiography or percutaneous coronary intervention. Although some studies included patients with existing renal dysfunction, recent meta-analyses show overall benefit of vitamin C in preventing CI-AKI compared to placebo or normal saline hydration [142,143]. An exact mechanism of reno-protection by ascorbate cannot be delineated from these analyses due to lack of available biochemical data. However, it is suggested that antioxidant ROS scavenging and vascular protection may predominate, largely based on animal studies of nephrotoxic AKI, and a human study showing that vitamin C exerted a positive change in total antioxidant status immediately after drug administration, and at follow-up [144]. Although a further recent study failed to show benefit of a standard dose of intravenous vitamin C in preventing CI-AKI in patients with chronic renal insufficiency, a post hoc analysis of the data did support a reduced rate of CI-AKI in patients with mildly impaired renal function [145].

#### 4.2.2. Benefit in Critical Illness and Sepsis

As well as providing benefit in cardiac surgery patients, vitamin C appears to benefit critically ill subjects with reduced new organ failure, ventilation, and/or time in ICU. In these studies, vitamin C was typically administered in combination with other micronutrients, vitamins E/B1, and/or selenium, so that its precise role was obscured [119,139]. However, in severe burn patients, a very high parenteral dose of vitamin C significantly reduced fluid requirements and improved urinary output, suggesting that early administration of vitamin C alone may improve morbidity in burn-induced shock [119]. Also, a recent phase I study of the safety of pharmacological doses of parenteral vitamin C, demonstrated significantly reduced multiple organ failure and pro-inflammation biomarkers in severe sepsis [146]. Low plasma vitamin C is common in patients with traumatic and critical illness, including sepsis and after cardiac surgery, and high intravenous dosages may be required to restore adequacy [119]. In a recent observational study of sepsis, early use of a combination of intravenous vitamin C, hydrocortisone, and thiamine, significantly reduced AKI, mortality, and progressive organ failure in septic patients [147]. In this study, vitamin C and hydrocortisone were proposed to act synergistically to preserve endothelium integrity and improve clinical outcomes.

Previous human studies relevant to renal injury support vitamin C producing beneficial effects on endothelium function. For example, a high intra-arterial dose of vitamin C improves endothelial-dependent vasodilatation after I/R injury and endotoxemia [148,149]. Also, vitamin C improves endothelium function and serum lipid oxidation in renal allograft transplant patients [150]. High-dose vitamin C supplementation in severe sepsis and shock may also positively benefit endogenous vasopressor synthesis via hydroxylase and monooxygenase enzymes that require ascorbate as a co-factor [151]. Vasopressors such as norepinephrine and vasopressin are important in regulating blood pressure and renal water retention in critically ill patients. An observational trial of vitamin C/hydrocortisone/thiamine supplementation in sepsis reported significant reduction in the use of vasopressors in patients receiving vitamin C [147].

Thus, in accordance with pre-clinical studies, vitamin C appears to be protective in pathologies relevant to human AKI via preserving endothelium and vascular function. Whether this benefit is attributed to direct ROS scavenging, or involves non-antioxidant functions, remains to be defined, but is important as ascorbate is a co-factor of various hydroxylase enzymes involved in vascular wall integrity and cell signaling processes. Regarding the latter, ascorbate controls HIF-1 activity by stabilizing its regulator prolyl hydroxylase, via maintaining the active site iron in a reduced (active) state. HIF-1 is a pro-survival transcription factor activated by limited oxygen, metabolic disturbance and oxidative stress, and may be important in preventing AKI via ischemic pre-conditioning [113]. However, over-activation of HIF-1 may be maladaptive in some pathologies [152] as intermittent hypoxia can mediate chronic ischemia-induced NOX expression to generate persistently elevated oxidative stress. Further, iNOS and some pro-inflammatory cytokines are activated by HIF-1 and NO^•^ can induce HIF-1 under non-limiting oxygen conditions (normoxia) such as inflammation [139,152]. Whether over-activation of HIF-1 contributes to renal injury in sepsis is largely unknown, however, ascorbate inhibits iNOS expression and activity in microvascular endothelial cells in vitro and in animal models of sepsis [140]. Thus, part of the mechanism whereby ascorbate shows efficacy in sepsis may also be via suppression of HIF-1-dependent genes.

Overall, whilst ROS and oxidative stress are closely linked to AKI, and this maybe a mechanism whereby ascorbate, as an antioxidant, intervenes, non-antioxidant bioactivities of vitamin C in immune and vascular function may contribute to its therapeutic action in renal injury and disease. Further studies are warranted to determine optimal dose and route of administration, as well as timing, e.g., in ischemic pre-conditioning, and to establish whether ascorbate supplementation is beneficial in cohorts with low vitamin C status, and if so, the precise mechanism of action.

## 5. Conclusions and Limitations

Despite advances in knowledge and treatment, AKI patients continue to have high mortality and morbidity, especially those with chronic medical conditions. Pre-clinical studies show that antioxidants alleviate renal injury and improve kidney function via reducing oxidative damage and/or inflammation, though several therapeutic antioxidants have largely failed to show benefit in human AKI. Vitamin C does appear to be efficacious in AKI in pathologies with endothelium dysfunction, or where low vitamin C predominates. The reno-protective effects of ascorbate may derive from its known antioxidant activity in scavenging source and derived ROS, including non-radical oxidants, and/or maintaining GSH for peroxidase activity, or BH_4_ for eNOS function. Ascorbate may also preserve vascular structure and microcirculatory flow independent of antioxidant function, via maintenance of Fe^2+^ and Cu^+^-containing hydroxylase and monooxygenase enzymes. The latter are essential in collagen and vasopressin synthesis central to vascular structure and functionality, and also modulate redox activated signaling pathways, such as HIF-1, down-regulating genes involved in pro-inflammation. Vitamin C shows promise as a reno-protectant in kidney injury, however, whether this is via its physiological role as an enzyme co-factor, or its recognized biochemical activity as an antioxidant, or both, remains to be fully defined.

## Figures and Tables

**Table 1:** Recent vitamin C intervention studies in animal models of ischemic, nephrotoxic and injurious AKI.

AKI Model	Vitamin C Dosage (mg/kg)	Renal Improvements			Proposed Mechanism	Ref.
		Oxidation/Inflammation	Antioxidants	Function/Damage		
**Single Therapy**						
Renal I/R in rats	250	↓ tissue lipid oxidation	↑ tissue GSH	↓ serum urea, creatinine, tubular damage, necrosis, casts	antioxidant inhibition of oxidative stress allows recovery of renal function	[81]
Renal I/R in rats	50, 100	↓ tissue lipid oxidation, O_2_^•−^, MPO	↑ tissue GSH, nitrate/nitrite	↓ serum urea, microproteinuria, urate improved creatinine clearance, anuria	activation of NO/soluble guanylyl cyclase pathway inhibitors reverse benefit	[82]
Aortic I/R in rats	50, 100	↓ tissue lipid oxidation, iNOS, MPO, IL-6	not determined	no effect on anuria partially improved microcirculation	reduces oxidative stress and inflammation	[83]
Renal I/R in mice	57	↓ renal artery ROS	↑ tissue GSH, NO, renal artery SOD	↓ serum urea, creatinine, renal artery resistance, tubular damage improved renal artery relaxation	O_2_^•−^ scavenging and regulation of SOD protects GSH/NO	[84]
RM in rats	20	↓ tissue lipid oxidation	↑ tissue SOD, catalase	no significant effect on urea, creatinine, GFR; trend to decrease iron accumulation, tubular necrosis, casts	ROS scavenging prevents formation of ferryl Mb	[85]
**Comparative Study**						
Renal I/R in rats	500	↓ tissue lipid oxidation, inflammation	↑ tissue catalase	↓ plasma urea, creatinine	Antioxidant > reno-protection than l-arginine	[86]
Renal I/R in rats	500	↓ tissue lipid oxidation, O_2_^•−^, MPO ↓ tissue inflammation	↑ tissue GSH, catalase	↓ serum urea, urate, tubular damage, casts, microproteinuria ↑ creatinine clearance	antioxidant, decreases O_2_^•−^ reno-protection similar to progesterone progesterone receptor antagonist reverses benefit	[87]
Remote organ I/R	100	↓ plasma & tissue lipid oxidation, inflammation	not determined	improved blood biochemistry (pO_2_, bicarbonate) ↓ tubular necrosis	vascular protective effects similar to synthetic prostacyclin	[88]
Nephrotoxicity in rats	100	↓ tissue lipid oxidation, inflammation	↑ tissue catalase, GSH, nitrite, serum antioxidants	↓ urea, creatinine, tubular necrosis improvements to serum protein	ROS scavenging decreases oxidative stress comparable to vitamin E	[89]
RM in rats	100	↓ plasma/tissue specific lipid oxidation, MCP-1, kinase activity (MAPK)	normalization of total & specific tissue GPx	↓ proteinuria, plasma urate, renal casts; normalisation of epithelial brush border	oxidative stress reduction comparable to a synthetic polyphenol; renal functional improvements unrelated to antioxidation	[77]
